# Targeting NOTCH1 in combination with antimetabolite drugs prolongs life span in relapsed pediatric and adult T-acute lymphoblastic leukemia xenografts

**DOI:** 10.1186/s40164-023-00439-6

**Published:** 2023-09-04

**Authors:** Sonia Minuzzo, Valentina Agnusdei, Marica Pinazza, Adriana A. Amaro, Valeria Sacchetto, Ulrich Pfeffer, Roberta Bertorelle, Orietta Spinelli, Valentina Serafin, Stefano Indraccolo

**Affiliations:** 1 Department of Surgery, Oncology and Gastroenterology, University of Padova, Padova, Italy; 2 Istituto Oncologico Veneto IOV - IRCCS, Padova, Italy; 3 Present Address: Becton Dickinson, Franklin Lakes, NJ 07417 USA; 4 IRCCS Ospedale Policlinico San Martino, Genova, Italy; 5 Hematology and Bone Marrow Transplant Unit of Azienda Ospedaliera Papa Giovanni XXIII, Bergamo, Italy

**Keywords:** T-ALL, Relapse, anti-NOTCH1, Antimetabolites

## Abstract

T-cell acute lymphoblastic leukemia (T-ALL) is a hematologic tumor, characterized by several genetic alterations, that constitutes 15% of pediatric and 25% of adult ALL. While with current therapeutic protocols children and adults’ overall survival (OS) rates reach 85–90% and 40–50%, respectively, the outcome for both pediatric and adult T-ALL patients that relapse or are refractory to induction therapy, remains extremely poor, achieving around 25% OS for both patient groups. About 60% of T-ALL patients show increased NOTCH1 activity, due to activating *NOTCH1* mutations or alterations in its ubiquitin ligase *FBXW7*. NOTCH signaling has been shown to contribute to chemotherapy resistance in some tumor models. Hence, targeting the NOTCH1 signaling pathway may be an effective option to overcome relapsed and refractory T-ALL.

Here, we focused on the therapeutic activity of the NOTCH1-specific monoclonal antibody OMP-52M51 in combination either with drugs used during the induction, consolidation, or maintenance phase in mice xenografts established from pediatric and adult relapsed *NOTCH1* mutated T-ALL samples. Interestingly, from RNAseq data we observed that anti-NOTCH1 treatment in vivo affects the purine metabolic pathway. In agreement, both in vitro and in vivo, the greatest effect on leukemia growth reduction was achieved by anti-NOTCH1 therapy in combination with antimetabolite drugs. This result was further corroborated by the longer life span of mice treated with the anti-NOTCH1 in combination with antimetabolites, indicating a novel Notch-targeted therapeutic approach that could ameliorate pediatric and adult T-ALL patients outcome with relapse disease for whom so far, no other therapeutic options are available.

**Supplementary Information:**

The online version contains supplementary material available at 10.1186/s40164-023-00439-6.

## To the editor

Thanks to the current therapeutic protocols, children and adults’ affected by T-cell acute lymphoblastic leukemia (T-ALL) [1] present an overall survival (OS) rate that reaches 85–90% and 40–50%, respectively [2, 3]. Nevertheless, around 25–40% of pediatric and adult T-ALL patients still experience relapses, with an OS around 25% for both patient groups [4, 5]. Furthermore, for relapsed T-ALL patients, except from Hematopoietic Stem Cell Transplantation and the intensification of the therapeutic regimen administered after the first diagnosis, no novel therapeutic options are available so far [1, 4, 5]. Therefore, the identification of novel therapeutic approaches are necessary to treat T-ALL relapsed patients thus preventing a poor outcome. In this light, *TP53* mutations and deletions have been shown to occur more frequently at relapse and are adversely associated with second-line therapy survival [6]. Additionally, 60% of T-ALL patients present activating *NOTCH1* mutations or alterations in its ubiquitin ligase *FBXW7* [7, 8], suggesting NOTCH1 signaling pathway as a possible therapeutic target to overcome relapsed T-ALL. In this regard, several preclinical studies have been reported either directly or indirectly inhibiting NOTCH1 signaling [9–11], but few reports on relapsed T-ALL treatment have been published so far [12]. Taking advantage of our previous studies [9] here we aimed to assess if NOTCH1 signaling inhibition by the specific monoclonal anti-NOTCH1 antibody (OMP-52M51) would be effective at relapse, exploiting NSG mice xenograft models established from both pediatric (PDTALL46, PDTALL39 and PDTALL47) and adult (PDTALL-AD2R and PDTALL-AD4) relapsed *NOTCH1* and *TP53* mutated T-ALL samples (Additional file 1: Table S1 and Fig. S1A-B).

**Fig. 1:**
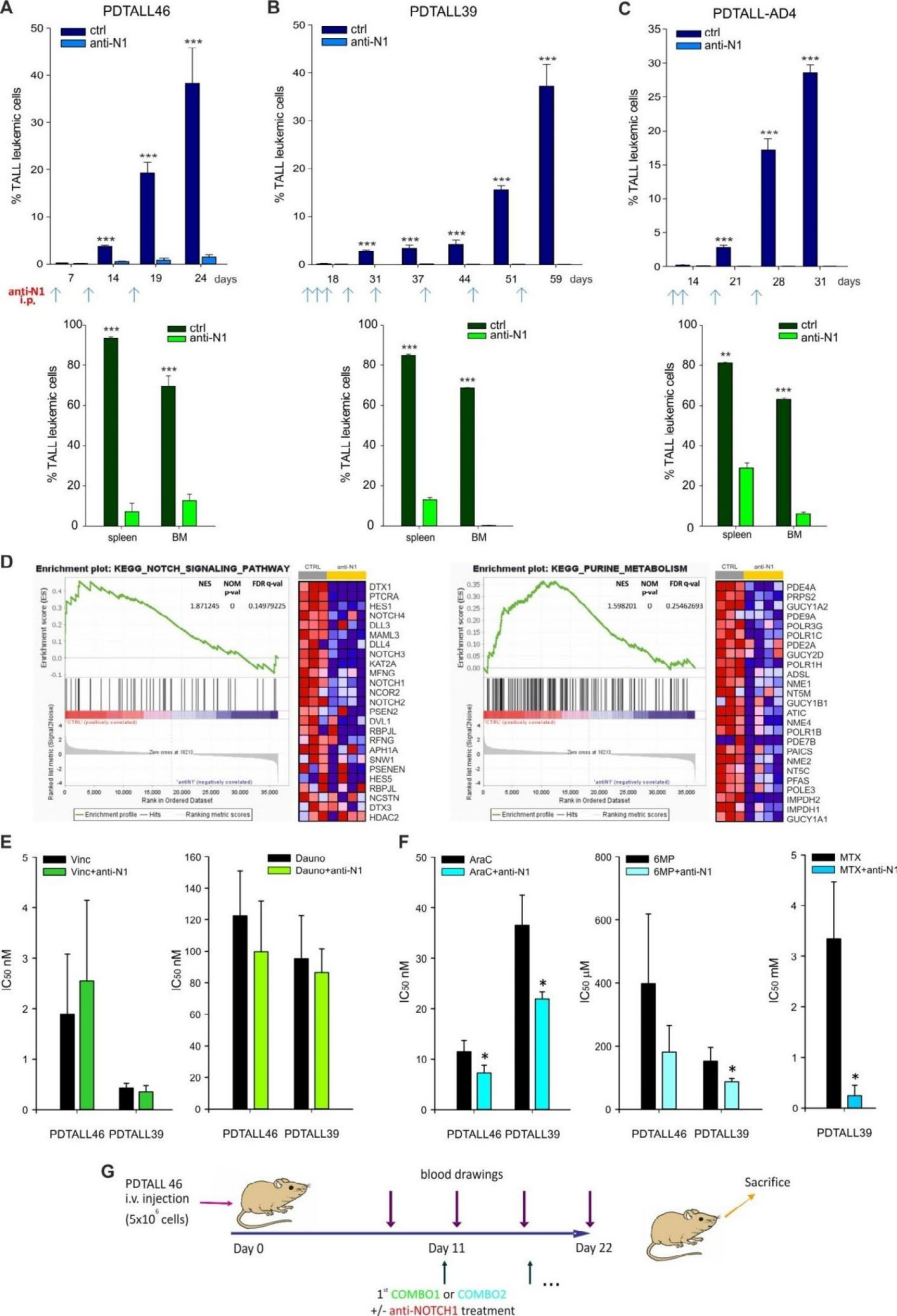
**A-C**: Anti-NOTCH1 (OMP-52M51) inhibits growth of NOTCH1-driven relapsed T-ALL PDXs. NSG mice (*n* = 5 mice/group) were i.p. treated with OMP-52M51 (anti-N1) or control antibody (ctrl Ab) at weekly intervals at 20 mg/Kg two days after i.v. injection of T-ALL cells (5 × 10^6^ cells/mouse) from 2 pediatric PDXs (A- PDTALL46, B- PDTALL39) and 1 adult PDXs (C- PDTALL-AD4). Antibodies injections are indicated by arrows. Top panels show leukemia engraftment by serial blood drawings and flow cytometric analysis of circulating blasts after first blood drawing, 7–19 days from the beginning of the experiment. The last blood drawing was obtained at sacrifice, when initial signs of illness appeared in control. Bottom panels display quantification of leukemia cells in the spleen and the BM at sacrifice. Statistically significant differences are indicated ** *P < 0.01;* *** *P < 0.001 t-test*. **D**: RNAseq analysis of OMP-52M51-acute treated PDTALL46 mice. Gene Set Enrichment Analysis (GSEA) plots and heat maps of the top 25 down-regulated differentially expressed genes in PDTALL46 anti-NOTCH1 OMP-52M51 (anti-N1) treated mice compared to mice treated with control antibody alone (3–4 samples/group). Red and blue indicate higher and lower expression levels, respectively. The columns represent individual samples. **E-F**: In vitro effect of OMP-52M51 (anti-N1) with antimetabolite drugs in T-ALL PDXs cells. Representation of IC_50_ values calculated for each chemotherapeutic drug alone or in combination with OMP-52M51 assessed by cleaved caspase-3/7 activity. IC_50_ values are expressed as the mean ± S.D. of at least three independent experiments. * *P < 0.05*. Values are reported in Additional file 1: Table S3. **G**: visualization of the therapeutic scheme to compare the in vivo efficacy of COMBO1 and COMBO2 drugs alone or in combination with anti-NOTCH1 antibody in PDTALL46 model

**Fig. 2:**
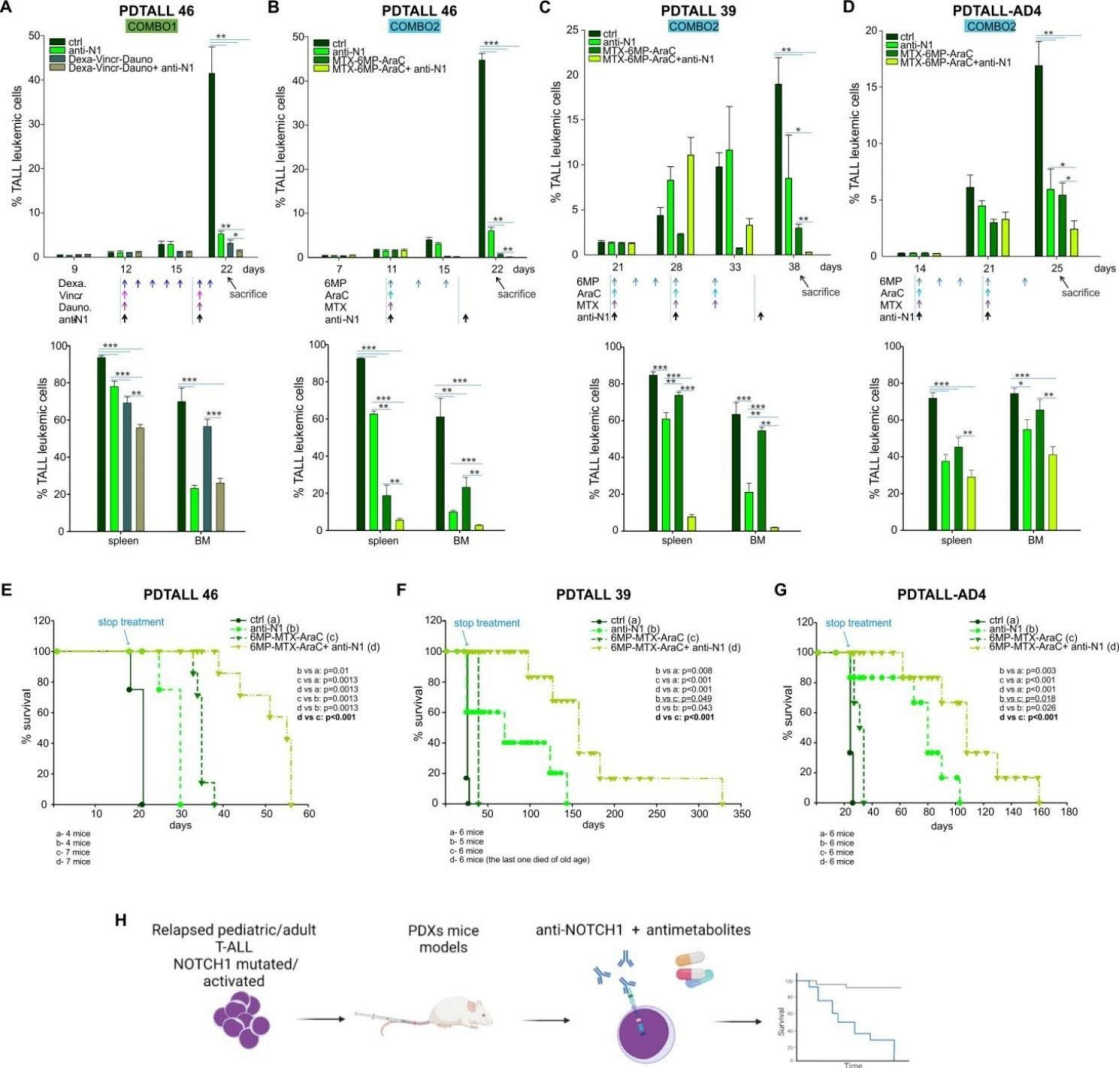
**A-B**: In vivo inhibitory effect of anti-NOTCH1 treatment in combination with chemotherapy in PDTALL46 model. Therapeutic effects of COMBO1 and COMBO2 chemotherapy alone or in combination with OMP-52M51 of PDTALL46 outgrowth in NSG mice. Top panels show the percentage of T-ALL cells in the blood of NSG mice at various time points. Bottom panels report the percentage of T-ALL cells in the spleen and bone marrow at sacrifice. (n = 5/6 per group). * P < 0.05; ** *P < 0.01;* *** *P < 0.001*. **C-D**: In vivo inhibitory effect of OMP-52M51 in combination with antimetabolite drugs (COMBO2) in the PDTALL39 and PDTALL-AD4 models. Therapeutic effects of COMBO2 chemotherapy (MTX-6MP-AraC) alone or in combination with OMP-52M51 of PDTALL39 (C) and PDTALL-AD4 (D) outgrowth in NSG mice. Top panels show the percentage of T-ALL cells in the blood of NSG mice at various time points. Bottom panels report the percentage of T-ALL cells in the spleen and bone marrow at sacrifice. Five to six mice per group were used. * P < 0.05; ** *P < 0.01;* *** *P < 0.001*. **E-G**: Anti-NOTCH1 in combination with antimetabolite drugs significantly increases survival in T-ALL PDXs models. Effect of COMBO2 chemotherapy alone or in combination with OMP-52M51 on survival of PDTALL46, PDTALL39 and PDTALL-AD4 injected NSG mice. **E-F-G** panels show Kaplan-Meier survival curves of PDTALL46, PDTALL39 and PDTALL-AD4 PDXs. Four to seven mice per group were used. Survival curves were compared by log-rank test. **H**: Visual representation of main results

As first, we treated PDX mouse models with anti-NOTCH1 monotherapy, started 2 days after i.v. injection of T-ALL relapse cells into mice, and we observed a clear leukemia burden reduction in the peripheral blood (PB) (Fig. 1A-C and Additional file 1: Fig. S2A upper panel), bone marrow (BM) and spleen (Fig. 1A-C and Additional file 1:  Fig. S2A bottom panel) in 4 out of 5 T-ALL PDXs. Only PDTALL-AD2R was apparently not responding to treatment (Additional file 1: Fig. S2B), probably due to the almost undetectable expression of *NOTCH1* target genes (Additional file 1: Fig. S1) suggesting the absence of a NOTCH1 pro-survival signaling dependence, despite the presence of a NOTCH1 PEST domain mutation. RNAseq analysis from in vivo PDTALL46 cells treated or not with OMP-52M51 unveiled that the anti-NOTCH1 therapy causes a significant down regulation of NOTCH1 signaling, histidine and tyrosine metabolism as well as purine metabolism which can be targeted by FDA-approved antimetabolites drugs used in T-ALL treatment (Fig. 1D, Additional file 1: Fig. S3 and Table S2). Accordingly, the in vitro apoptosis Caspase 3/7 assay on PDTALL46 and PDATALL39 primary T-ALL cells revealed the most significant IC_50_ index decrease in the combination between anti-NOTCH1 and antimetabolites used during the consolidation/maintenance phases [Cytarabine (AraC), methotrexate (MTX) and 6-mercaptopurine (6MP)], (Fig. 1F, Additional file 1:  Fig. S4 and Table S3), compared to drugs administered along the induction phase therapy [vincristine (Vinc) and daunorubicin (Dauno)] (Fig. 1E). Thus, starting from these results and based on the kinetics of PDTALL46 leukemia growth (Additional file 1: Fig. S5A), we started the in vivo treatment (day 11) with the anti-NOTCH1 alone or in combination with COMBO1 (Vinc, Dauno, Dexa) or COMBO2 (AraC, MTX, 6MP) schedule when the percentage of CD5^+^/CD7^+^ circulating blasts in the PB of PDTALL46 was around 1–2% (Additional file 1: Table S4, Fig. 1G). Interestingly, we observed a significant reduction of CD5^+^/CD7^+^ blasts in mice treated with the anti-NOTCH1 antibody in combination with both therapeutic schedules (COMBO1/2) in all the compartments (PB, BM and spleen) as well as a decrease in spleen weight (Additional file 1: Fig. S5B) compared to controls or single arm treatments (Fig. 2A-B). Importantly, mice treated with both the anti-NOTCH1 antibody and COMBO2 showed the best therapeutic effect (Fig. 2B). These results were further confirmed in the pediatric PDTALL39 (Fig. 2C and Additional file 1: Fig. S6A) and in the adult PDTALL-AD4 (Fig. 2D and Additional file 1: Fig. S6B) PDX models, although in the latter with less efficacy when compared to the pediatric one, probably due to the fact that adult T-ALL have lower response rate to chemotherapy and thus result more difficult to treat.

Finally, we performed survival experiments by administrating the anti-NOTCH1 and COMBO2 treatments alone or in combination, and stopped the treatments at 20-40% of circulating blasts in control mice PB (Additional file 1: Fig. S7A-C). In agreement with the efficacy in vivo studies, all PDX mice models treated with the anti-NOTCH1 and COMBO2 therapy showed a significantly (p < 0.001) longer life span survival, between 20 and 290 days, compared to the COMBO2 alone treated group (0-100 days) (Fig. 2E-G), thus corroborating the hypothesis that NOTCH1 targeted therapy improves therapeutic efficacy of antimetabolite drugs (Fig. 2H).

In conclusion, altogether these results provide a rationale for a novel therapeutic strategy that provides NOTCH1 inhibition in combination with antimetabolites drugs in T-ALL relapsed pediatric and adult patients, for whom so far no other therapeutic options are available.

### Electronic supplementary material

Below is the link to the electronic supplementary material.

**Additional file 1: Additional materials and methods.** Establishment of T-ALL xenografts and treatments. Sanger sequencing. Reverse transcription-PCR (RT-PCR) and quantitative PCR (qPCR). Western blot analysis. Preparation of RNA libraries and RNA seq. Bioinformatics analysis. T-ALL Patient Derived Xenografts (PDXs) cells in vitro treatments and (half maximal inhibitory concentration) IC50 determination for selected drugs by Caspase-Glo® 3/7 Assay. Statistical analysis. Scientific Image and Illustration software. **Additional Tables: Table S1.** Characterization of PDXs from T-ALL pediatric and adult relapsed patients. **Table S2.** Upregulated gene sets in CTRL versus OMP-52M51-treated PDTALL46 cells. **Table S3.** IC50 determination for selected drugs in T-ALL PDX cells. **Table S4.** Selected drugs to be combined with anti-NOTCH1 antibody (OMP-52M51). **Additional Figures and Legends:** **Figure S1.** NOTCH1 protein and target genes expression in T-ALL PDXs (PDTALL). **Figure S2. **Anti-NOTCH1 (OMP-52M51) inhibits growth of NOTCH1-driven T-ALL PDXs. **Figure S3.** RNASeq analysis of OMP-52M51-acute treated PDTALL46 mice. **Figure S4.** In vitro cell apoptosis determination in T-ALL PDXs cells treated with different drugs alone or in combination with OMP-52M51. **Figure S5.** In vivo inhibitory effect of OMP-52M51 in combination with COMBO1 and COMBO2 in PDTALL46 model. **Figure S6.** In vivo inhibitory effect of OMP-52M51 in combination with antimetabolite drugs (COMBO2) in PDTALL39 and PDTALL-AD4 models. **Figure S7.** Efficacy of Anti-NOTCH1 in combination with antimetabolite drugs in T-ALL PDXs models. **Additional References.**

## Data Availability

RNAseq data have been deposited in NCBI’s Gene Expression Omnibus and are accessible through GEO accession number GSE224988.
