# Supplementary material for: Targeting NOTCH1 in combination with antimetabolite drugs prolongs life span in relapsed pediatric and adult T-acute lymphoblastic leukemia xenografts
Source: Exp Hematol Oncol. 2023 Sep 4;12:76. doi: 10.1186/s40164-023-00439-6 (PMC10476325; doi:10.1186/s40164-023-00439-6)
Supplement: Supplementary file 1 — Additional file 1: Additional materials and methods. Establishment of T-ALL xenografts and treatments. Sanger sequencing. Reverse transcription-PCR (RT-PCR) and quantitative PCR (qPCR). Western blot analysis. Preparation of RNA libraries and RNA seq. Bioinformatics analysis. T-ALL Patient Derived Xenografts (PDXs) cells in vitro treatments and (half maximal inhibitory concentration) IC50 determination for selected drugs by Caspase-Glo® 3/7 Assay. Statistical analysis. Scientific Image and Illustration software. Additional Tables: Table S1. Characterization of PDXs from T-ALL pediatric and adult relapsed patients. Table S2. Upregulated gene sets in CTRL versus OMP-52M51-treated PDTALL46 cells. Table S3. IC50 determination for selected drugs in T-ALL PDX cells. Table S4. Selected drugs to be combined with anti-NOTCH1 antibody (OMP-52M51). Additional Figures and Legends: Figure S1. NOTCH1 protein and target genes expression in T-ALL PDXs (PDTALL). Figure S2. Anti-NOTCH1 (OMP-52M51) inhibits growth of NOTCH1-driven T-ALL PDXs. Figure S3. RNASeq analysis of OMP-52M51-acute treated PDTALL46 mice. Figure S4. In vitro cell apoptosis determination in T-ALL PDXs cells treated with different drugs alone or in combination with OMP-52M51. Figure S5. In vivo inhibitory effect of OMP-52M51 in combination with COMBO1 and COMBO2 in PDTALL46 model. Figure S6. In vivo inhibitory effect of OMP-52M51 in combination with antimetabolite drugs (COMBO2) in PDTALL39 and PDTALL-AD4 models. Figure S7. Efficacy of Anti-NOTCH1 in combination with antimetabolite drugs in T-ALL PDXs models. Additional References. [file 40164_2023_439_MOESM1_ESM.docx]

**SUPPLEMENTARY MATERIALS AND METHODS**

**Establishment of T-ALL xenografts and treatments**

Xenografts (PDTALL) establishment has been previously reported^1^ and their genetic status of *NOTCH1*, *FBXW7*, and *TP53* is reported in Table S1. PDTALL cells were intravenously (i.v.) injected in NSG mice (5×10^6^ cells/mouse; 5-6 mice/group) and animals were intraperitoneally (i.p.) treated with the humanized anti-human NOTCH1 monoclonal antibody OMP-52M51 (Oncomed Pharmaceuticals Inc., Redwood, CA, USA) or control antibody (Rituximab, Roche, Basel, Switzerland) alone or in combination with the chemotherapeutic regimen reported in Table S4. Control group also received the vehicle indicated in column “Injection buffer” in Table S4. Dosage and time of treatment were established based on literature data^2-5^. Treatments started when leukemia was engrafted. The timing of treatment was slightly different in the various xenograft models and based on the different kinetics of leukemia development.

In the acute treatment experiment, PDTALL46 xenografts were treated with anti-NOTCH1 or control antibody 4 days before sacrifice.

Procedures involving animals conformed to current laws and policies (EEC Council Directive 2010/63/EU, OJ L 276, 20.10.2010) were authorized by the Italian Ministry of Health (894/2016-PR). T-ALL growth was monitored by periodic blood drawings and flow cytometric analysis of CD5 and CD7 (the antibodies used were both from Coulter, Fullerton, CA, USA). Leukemic cells were recovered from the spleen and used for the following analyses.

**Sanger sequencing**

DNA was amplified by Titanium® DNA Amplification Kit (Clontech, Saint-Germain-en-Laye France) and sequenced by BigDye® Terminator v1.1 Cycle sequencing Kit (Applied Biosystems). Primers used are:

NOTCH1ex27_for: CATGGGCCTCAGTGTCCT

NOTCH1ex27_rev: TAGCAACTGGCACAAACAGC

NOTCH1ex28_for: GCGTAGCCGCTGCCTGAT

NOTCH1ex28_rev: CAGACTCCCGGTGAGGATGC

NOTCH1ex34T_for: GCTGGCCTTTGAGACTGG

NOTCH1ex34T_rev: CTCCTGGGGCAGAATAGTGT

NOTCH1ex34P_for: ACAGATGCAGCAGCAGAACC

NOTCH1ex34P_rev: CCTGGGGCCAGATAAAACAGTACA

Primer sequences for NOTCH1ex26 and FBXW7 sequencing are reported in our previous paper^6^. *TP53* *locus* was sequenced as previously described^7^.

### Reverse transcription-PCR (RT-PCR) and quantitative PCR (qPCR)

### Total RNA was isolated using TRIzol Reagent according to the manufacturer’s instructions. cDNA was synthesized from 1-1.5 μg of total RNA using the High Capacity RNA-to-cDNA kit. For qPCR analysis, the SYBR Green dye and ABI Prism 7900 Sequence Detection System were used. All reagents were obtained from Life Technologies, Paisley, UK. Relative quantification was done using the DDCt method, normalizing to β2-microglobulin mRNA. Primer sequences for *CR2*, *HES1*, *DTX1*, *PTCRA* and *NOTCH3* were previously reported^1^.

###

###

### Western blot analysis

Western blot methods used in this study have been previously published^6^. Immunoprobing was performed using anti-NOTCH1 full-length (FL) and intra cellular domain (ICD)-Val1744 (both from Cell Signaling Technology, Danvers, MA, USA) and anti-Actin (Merck Life Science, Milan, Italy) antibodies. Antigens were identified by luminescent visualization using the Western Lightning Plus ECL (Perkin Elmer, Waltham, MA, USA) or ECL Select (Amersham, GE Healthcare, Chicago, IL, USA).

**Preparation of RNA libraries and RNA seq**

Total RNA was isolated from PDTALL46 cells of spleens of 4 control and 4 anti-N1 Ab-treated mice (acute treatment), using TRIzol Reagent according to the manufacturer’s instructions. The 8 RNAs were treated with DNasi (Ambion) and checked for quality (Agilent Bioanalyzer, Agilent) before proceeding to RNA libraries preparation. The 8 RNAseq libraries were produced using the NEB-Next rRNA Depletion Kit (NEB). All samples were sequenced in 150 paired-end with an Illumina platform. Libraries preparation and RNAseq were performed by Personal Genomics SRL (Verona, Italy).

**Bioinformatics analysis**

Quality check of the raw sequencing reads was performed using FastQC (v0.11.9). Quality filtering and check of the reads were performed using Fastp (v0.20.1) with the following set of parameters: length_required=36, cut_right, cut_right_window_size=4, cut_right_mean_quality=15, trim_poly_g, overrepresentation_analysis. Potential mouse contaminating reads were removed by competitive mapping to the human/mouse hybrid genome using BBSplit (BBMap v38.84). The alignment of high-quality human reads on the reference genome Gencode GRCh38 release 37 was performed with STAR (v2.7.8a), using the basic annotation gtf file. RNASeq data have been deposited in NCBI’s Gene Expression Omnibus (5) and are accessible through GEO accession number GSE224988.

Gene expression quantification was carried out using Stringtie (v2.1.5). Read counts for each gene and transcript were derived from the coverage values estimated by StringTie for each sample using Python script prepDE.py, provided with Stringtie. Gene expression counts were imported into DESeq2 (v1.22.2) R package for normalization using DESeq2’s median of ratios method and differential expression analysis (Wald test). The analysis was performed by comparing 3 T-ALL specimens from mice treated with ctrl antibody versus 4 T-ALL samples from mice treated with the anti-NOTCH1 monoclonal antibody. One control sample was excluded from the analysis because it did not pass the quality check. The differentially expressed genes for each comparison were selected using an adjusted p-value < 0.05. The computational method of gene set enrichment analysis was performed on RNASeq data in order to find groups of genes that are statistically over-represented in a large set of genes collected in Gene Ontology or Kegg pathways and may have an association with disease phenotypes. Genes are ranked based on the correlation between their expression and the class of the analyzed samples. GSEA (Gene set enrichment analysis) software is available at <http://software.broadinstitute.org/gsea/index.jsp>^8-9^. We searched for significantly associated gene sets from one or both categories Control and OMP-52M51 (anti-N1) antibodies. SDC, Table 2 lists upregulated in Control *versus* OMP-52M51 (anti-N1) antibody gene sets with an FDR ≤ 0.25.

**T-ALL Patient Derived Xenografts (PDXs) cells *in vitro* treatments and (half maximal inhibitory concentration) IC_50_ determination for selected drugs by Caspase-Glo® 3/7 Assay**

Cells from spleen of two different T-ALL PDXs were treated with the drugs listed in Table S4, alone or in combination with OMP-52M51, to perform an *in vitro* cell death assay to determine the dose that induces apoptosis in 50% cells (IC50). Briefly, 10^5^ cells/well from PDTALL46 and PDTALL39 were seeded in 96-well plates and immediately treated with serial compound dilutions for 24-72 h, alone or in combination with OMP-52M51 at the constant concentration of 10 μg/ml. Different concentrations of OMP-52M51 alone did not induce TALL cell death (upper left panel Figure S4 A-B).

IC50 values were determined with the Caspase-Glo® 3/7 Assay (Promega, Italy), evaluating cleaved caspase-3/7 activity, by the addition of 100 μl of reagent at the end of treatment and calculated using the CompuSyn software (Biosoft, Cambridge, United Kingdom).

**Statistical analysis**

Results were expressed as mean value± S.D. Statistical analysis of data was performed using Student’s t-test, when samples followed a normal distribution, or non-parametric Mann–Whitney–Wilcoxon test. Mouse survival was calculated using the Kaplan-Meier method, and survival curves were compared by log-rank test. Differences were considered statistically significant when *P<0.05*.

**Scientific Image and Illustration software**

Image in Figure 2H was created with BioRender.com.

**Table S1. Characterization of PDXs from T-ALL pediatric and adult relapsed patients.**

| **RELAPSE PDX** | **Pediatric/Adult** | **Phenotype** | **PGR/PPR** | ***N1* status** | ***N1-***  ***Ex26*** | ***N1-***  ***Ex27*** | ***N1-***  ***Ex28*** | ***N1-***  ***Ex34 TAD*** | ***N1-Ex34 PEST*** | ***FBXW7* status** | ***FBXW7-***  ***Ex9*** | ***FBXW7-***  ***Ex 10*** | **P53 status** |
| --- | --- | --- | --- | --- | --- | --- | --- | --- | --- | --- | --- | --- | --- |
| **PDTALL39** | Pediatric | Thym | PPR | **HD** | T4754C => L1585P (het): COSM13046 pathogenic | wt | wt | wt | wt | wt | wt | wt | **p.P190T** |
| **PDTALL46** | Pediatric | Early T | PPR | **HD** | 4747 Ins CCG => 1583 Ins P (het) | wt | wt | wt | wt | **mut** | wt | C1435T => R479*Stop: COSM206697 pathogenic | **p.R248Q** |
| **PDTALL47** | Pediatric | Early T | / | **HD** | T4799C => L1600P (het): COSM12771 pathogenic | wt | wt | wt | wt | **mut** | G1394A=> R465H (het): COSM22965 pathogenic | wt | **p.R213Q**  **p.M237fs** |
| **PDTALL-**  **AD2R** | Adult | T | / | **PEST (hom)** | wt | wt | wt | wt | Hom: 7541 del CT; 2514RVP*STOP: COSM12774 | wt | wt | wt | **p.G262D** |
| **PDTALL-**  **AD4** | Adult | T | / | **HD** | 4819 ins CTCAGCCCTGTC => p. F1606_K1607 ins12 [LSPV] (het) | wt | wt | wt | wt | **mut** | G1394A=> R465H (het): COSM22965 pathogenic | wt | **p.S240R** |

PGR: Prednisone good responder; PPR: Prednisone poor responder; N1: NOTCH1

**Table S2. Upregulated gene sets in CTRL versus OMP-52M51-treated PDTALL46 cells.**

| **NAME** | **SIZE** | **ES** | **NES** | **NOM p-val** | **FDR q-val** | **FWER p-val** |
| --- | --- | --- | --- | --- | --- | --- |
| KEGG_NOTCH_SIGNALING_PATHWAY | 47 | 0,456495 | 1,871245 | 0 | 0,14979225 | 0,103 |
| KEGG_HISTIDINE_METABOLISM | 24 | 0,6643 | 1,65315 | 0 | 0,1915085 | 0,558 |
| KEGG_B_CELL_RECEPTOR_SIGNALING_PATHWAY | 73 | 0,265918 | 1,673049 | 0 | 0,2070863 | 0,501 |
| KEGG_GLYCOSAMINOGLYCAN_BIOSYNTHESIS_KERATAN_SULFATE | 15 | 0,5886 | 1,764665 | 0 | 0,2220301 | 0,33 |
| KEGG_HEMATOPOIETIC_CELL_LINEAGE | 73 | 0,355735 | 1,619383 | 0 | 0,23252171 | 0,606 |
| KEGG_TYROSINE_METABOLISM | 34 | 0,411632 | 1,598201 | 0 | 0,25462693 | 0,72 |
| KEGG_PURINE_METABOLISM | 140 | 0,361912 | 1,675855 | 0 | 0,26511502 | 0,501 |
| KEGG_GLYCOLYSIS_GLUCONEOGENESIS | 48 | 0,421779 | 1,547822 | 0 | 0,29790947 | 0,816 |
| KEGG_BASE_EXCISION_REPAIR | 34 | 0,532344 | 1,529912 | 0 | 0,29942888 | 0,918 |
| KEGG_AMINOACYL_TRNA_BIOSYNTHESIS | 41 | 0,552291 | 1,508856 | 0 | 0,31113958 | 0,948 |
| KEGG_WNT_SIGNALING_PATHWAY | 131 | 0,239671 | 1,35127 | 0 | 0,33530638 | 0,972 |
| KEGG_OLFACTORY_TRANSDUCTION | 118 | 0,408019 | 1,335448 | 0 | 0,3399016 | 1 |
| KEGG_ONE_CARBON_POOL_BY_FOLATE | 17 | 0,596163 | 1,484939 | 0 | 0,34556141 | 0,972 |
| KEGG_METABOLISM_OF_XENOBIOTICS_BY_CYTOCHROME_P450 | 35 | 0,422175 | 1,363258 | 0 | 0,35081962 | 0,972 |
| KEGG_RNA_DEGRADATION | 56 | 0,424576 | 1,327544 | 0 | 0,35198796 | 1 |
| KEGG_CELL_ADHESION_MOLECULES_CAMS | 95 | 0,367845 | 1,29501 | 0 | 0,36473265 | 1 |
| KEGG_ARGININE_AND_PROLINE_METABOLISM | 51 | 0,453408 | 1,416393 | 0 | 0,36697474 | 0,972 |
| KEGG_GLYCOSAMINOGLYCAN_BIOSYNTHESIS_HEPARAN_SULFATE | 23 | 0,420952 | 1,461839 | 0 | 0,36853105 | 0,972 |
| KEGG_O_GLYCAN_BIOSYNTHESIS | 26 | 0,416818 | 1,424371 | 0 | 0,37008616 | 0,972 |
| KEGG_ASTHMA | 15 | 0,587847 | 1,377572 | 0 | 0,3764375 | 0,972 |
| KEGG_DRUG_METABOLISM_CYTOCHROME_P450 | 36 | 0,44948 | 1,391837 | 0 | 0,3998288 | 0,972 |

96/167 gene sets are upregulated in CTRL-treated PDTALL46 cells (3 samples) and downregulated in anti-Notch1-treated cells (4 samples). 6 gene sets are significant at FDR < 25% and 21 gene sets are significantly enriched at nominal p value < 1%.

**Table S3. IC50 determination for selected drugs in T-ALL PDX cells.**

|  | **DRUG** | **PDTALL46** | **PDTALL39** |
| --- | --- | --- | --- |
|  |  | IC50 (24h) | IC50 (24/72h) |
|  |  |  |  |
| **COMBO1** | Dexa | n.d. (refractory) | n.d. (refractory) |
|  | Dexa+OMP-52M51 | n.d. | n.d. |
|  |  |  |  |
|  | Vinc | 1.89 ± 1.19 nM | 0.42 ± 0.1 nM |
|  | Vinc+OMP-52M51 | 2.55 ± 1.60 nM | 0.35 ± 0.13 nM |
|  |  | (refractory) |  |
|  | Dauno | 122.61 ± 28.16 nM | 97.2 ± 27.5 nM |
|  | Dauno+OMP-52M51 | 99.83 ± 32.1 nM | 86.5 ± 14.98 nM |
|  |  |  |  |
| **COMBO2** |  |  |  |
|  | AraC | **11.41 ± 2.25 nM** | **36.51 ± 5.95 nM** |
|  | AraC+OMP-52M51 | **7.31 ± 1.46 nM** | **21.95 ± 1.34 nM** |
|  |  |  |  |
|  | MTX | refractory | **3.34 ± 1.1 mM** |
|  | MTX+OMP-52M51 | refractory | **0.25 ± 0.2 mM** |
|  |  |  |  |
|  | 6MP | 397.5 ± 229.9 μM | **152.1 ± 43.8 μM** |
|  | 6MP+OMP-52M51 | 180.1 ± 85.8 μM | **86.5.29 ± 9.96 μM** |
|  |  |  |  |

Dose to induce apoptosis in 50% cells (IC_50_) was calculated by Compusyn software in 2 different T-ALL PDXs (PDTALL46 and PDTALL39), after 24/72h treatment with the indicated drugs, alone or in combination with the anti-NOTCH1 antibody OMP-52M51 (10 μg/ml). Statistically significant values (*P<0.05*) are highlighted in bold.

**Table S4. Selected drugs to be combined with anti-NOTCH1 antibody (OMP-52M51).**

| **Drug** | **Company** | **Action** | **Concentration** | **Dosage** | **Administration/**  **Volume** | **Injection buffer** | **Treatment schedule** |
| --- | --- | --- | --- | --- | --- | --- | --- |
| **Dexamethasone** | **Sigma-Aldrich, Inc. (D1756)** | **receptor mediated lympholysis** | **25 mg/ml in DMSO** | **5 mg/kg** | **i.p./200µl** | **H_2_O/PEG** | **5 x week** |
| **Vincristine** | **Teva Italia Srl** | **mitotic inhibitor** | **1 mg/ml** | **0.05 mg/kg** | **i.p./200µl** | **H_2_O/PEG** | **1 x week** |
| **Daunorubicin**  **(Daunoblastina®)** | **Pfizer Italia Srl** | **DNA intercalation** | **2 mg/ml** | **2.5 mg/kg** | **i.v./200µl** | **PBS** | **1 x week** |
| **Methotrexate** | **Pfizer Italia Srl** | **antimetabolite (DNA/RNA synthesis inhibitor)** | **25 mg/ml** | **1 mg/kg** | **i.p./200µl** | **H_2_O/PEG** | **1 x week** |
| **6-Mercaptopurine** | **Molekula Srl (1265909)** | **antimetabolite (DNA synthesis inhibitor)** | **50 mg/ml in DMSO** | **50 mg/kg** | **i.p./200µl** | **H_2_O/PEG** | **3 x week** |
| **Cytarabine** | **Accord Healthcare Italia Srl** | **antimetabolite (DNA synthesis inhibitor)** | **100 mg/ml** | **25 mg/kg** | **i.p./200µl** | **H_2_O/PEG** | **1 x week** |
| **Rituximab (MabThera)** | **Roche Ltd** | **chimeric anti-human CD20 monoclonal antibody** | **10 mg/ml** | **20 mg/kg** | **i.p./200µl** | **PBS** | **1 x week** |
| **anti-Notch1 (OMP-52M51)** | **OncoMed Pharmaceutical,Inc.** | **humanized anti-human NOTCH1 monoclonal antibody** | **10 mg/ml** | **20 mg/kg** | **i.p./200µl** | **PBS** | **1 x week** |

Dexamethasone+Vincristine+Daunorubicin = COMBO1; Methotrexate+6-Mercaptopurine+Cytarabine = COMBO2

COMBO1 is composed of drugs used for treatment of TALL during the induction phase, whereas COMBO2 consists of antimetabolite drugs used during the consolidation/maintenance phases. In all the experiments with the humanized anti-NOTCH1 monoclonal antibody OMP-52M51, the chimeric anti-human CD20 monoclonal antibody Rituximab is used as control antibody, since TALL cells do not express CD20.

**Figure S1.**

**
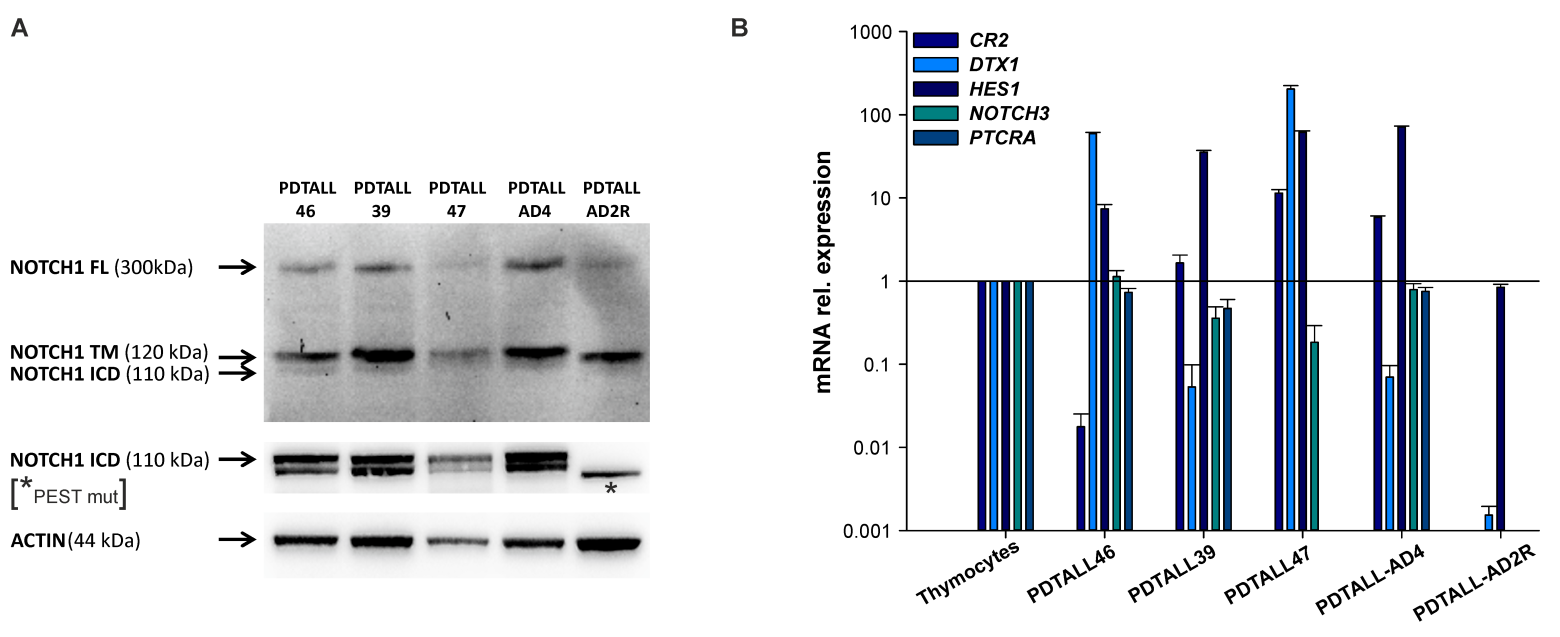
**

**NOTCH1 protein and target genes expression in T-ALL PDXs (PDTALL)**

A: Western Blot analysis of NOTCH1 protein levels in PDTALL xenografts (FL: Full-Length, TM: TransMembrane, ICD: Intra Cellular Domain). B: Analysis of *CR2, DTX1, HES1, NOTCH3* and *PTCRA NOTCH*-target genes expression by qRT-PCR in PDTALL xenografts. Expression data are referred to three biological replicates and normalized on Thymocytes as reference (set at 1). Data are reported as 2^-ΔCt^ (ΔCt= Ct gene- Ct β2-microglobulin, the housekeeping gene).

**Figure S2.**


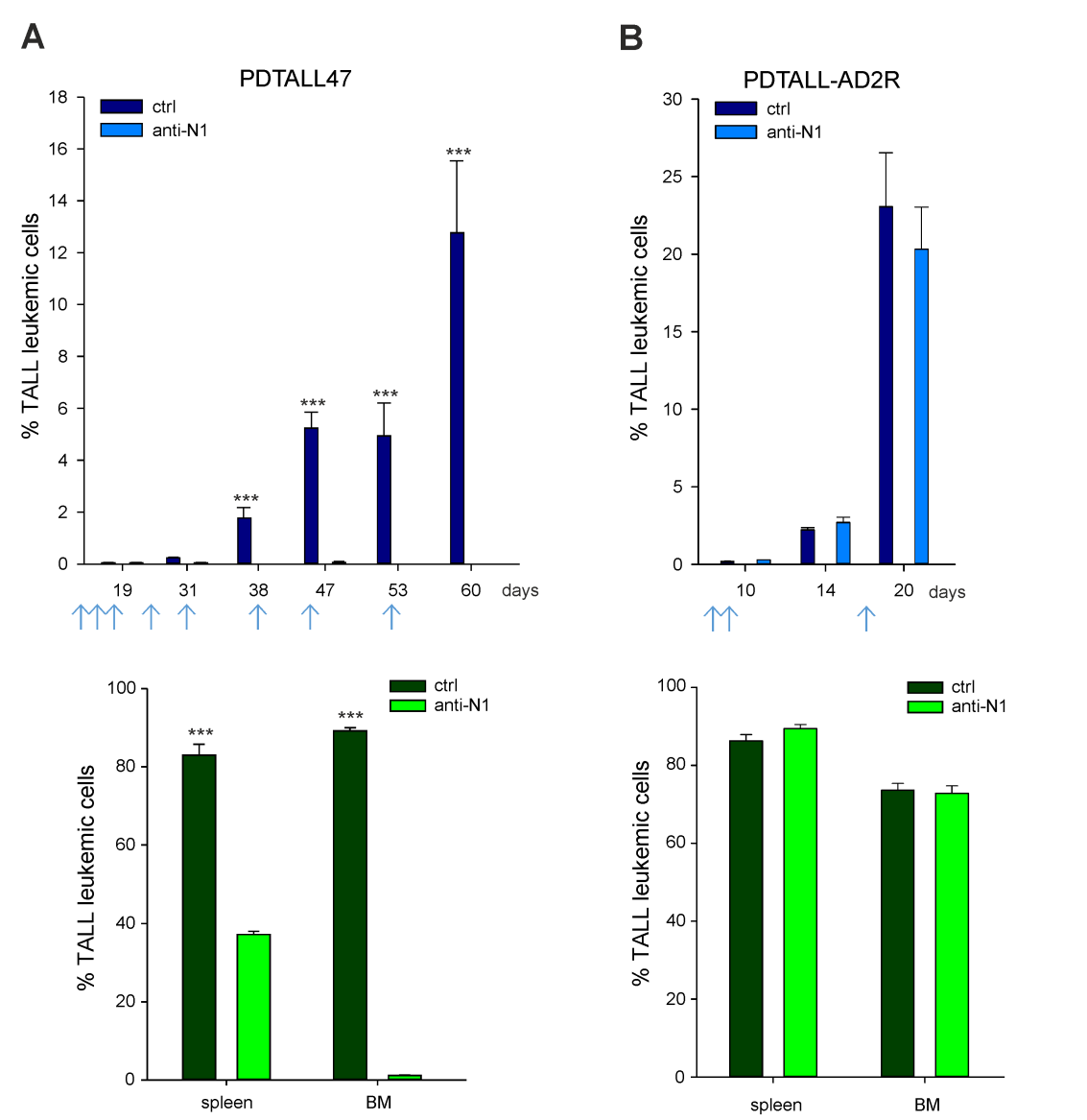


**Anti-NOTCH1 (OMP-52M51) inhibits growth of NOTCH1-driven T-ALL PDXs**

A-B. NSG mice (*n*=5 mice/group) were i.p. treated with OMP-52M51 (anti-N1) or control antibody (ctrl Ab) at weekly intervals at 20 mg/Kg two days after i.v. injection of T-ALL cells (5x10^6^ cells/mouse) from 1 pediatric PDX (A- PDTALL47) and 1 adult PDX (B). Antibodies injections are indicated by arrows.

Top panels show leukemia engraftment by serial blood drawings and flow cytometric analysis of circulating blasts after first blood drawing, 7-19 days from the beginning of the experiment. The last blood drawing was obtained at sacrifice, when initial signs of illness appeared in control. Bottom panels display quantification of leukemia cells in the spleen and the BM at sacrifice. Statistically significant differences are indicated (*** *P<0.001 t-test*).

**Figure S3.**


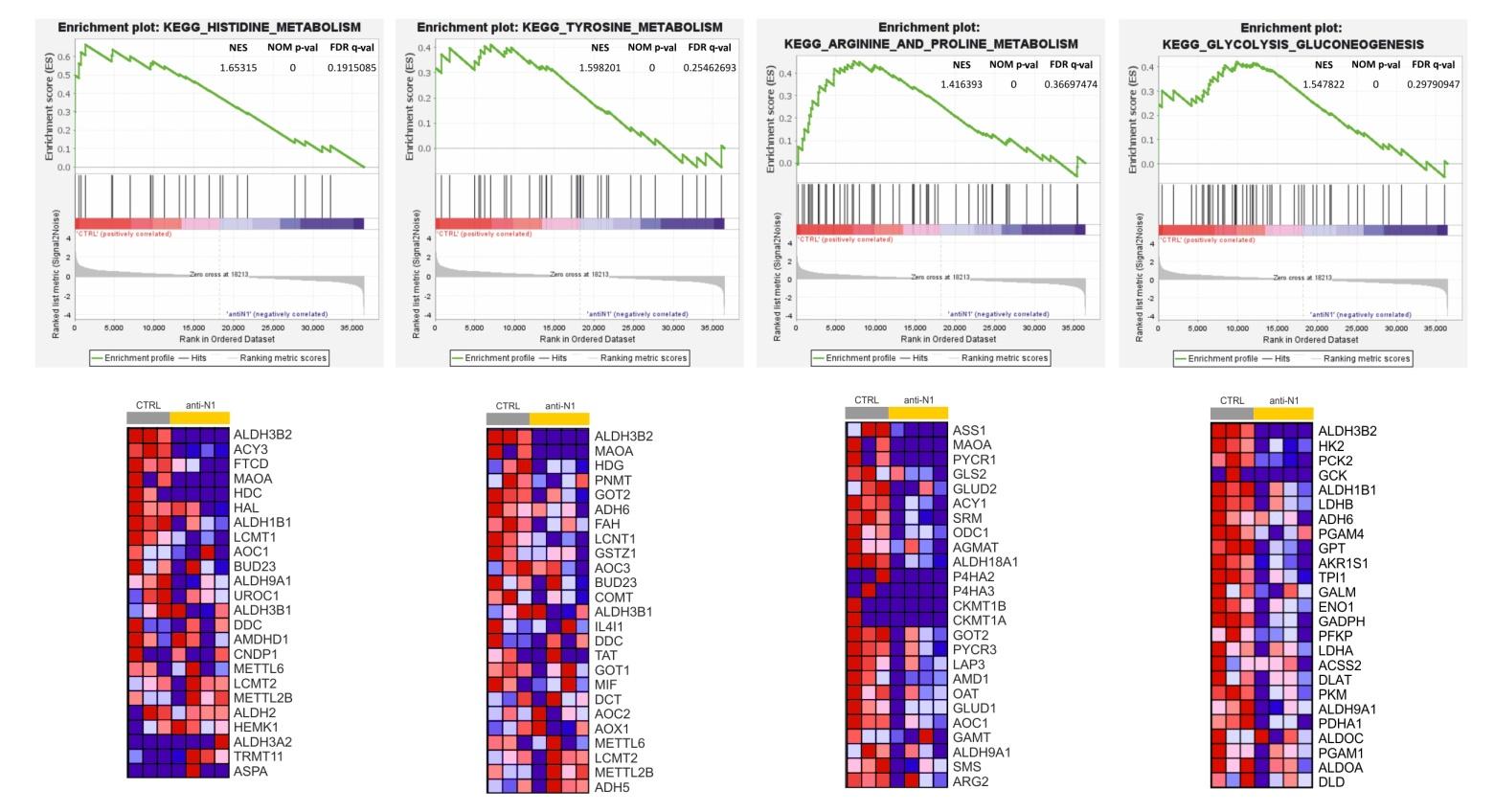


**RNASeq analysis of OMP-52M51-acute treated PDTALL46 mice**

Top: GSEA plots of four enrichment sets down-regulated in anti-Notch1-acute treated mice. Bottom: Heat maps and lists of the top 25 differentially expressed genes of the corresponding plots reported above and comparing TALL cells from mice treated with control and OMP-52M51 (anti-N1) antibody (3-4 samples/group). Red and blue indicate higher and lower expression levels, respectively. The columns represent individual samples.

**Figure S4.**


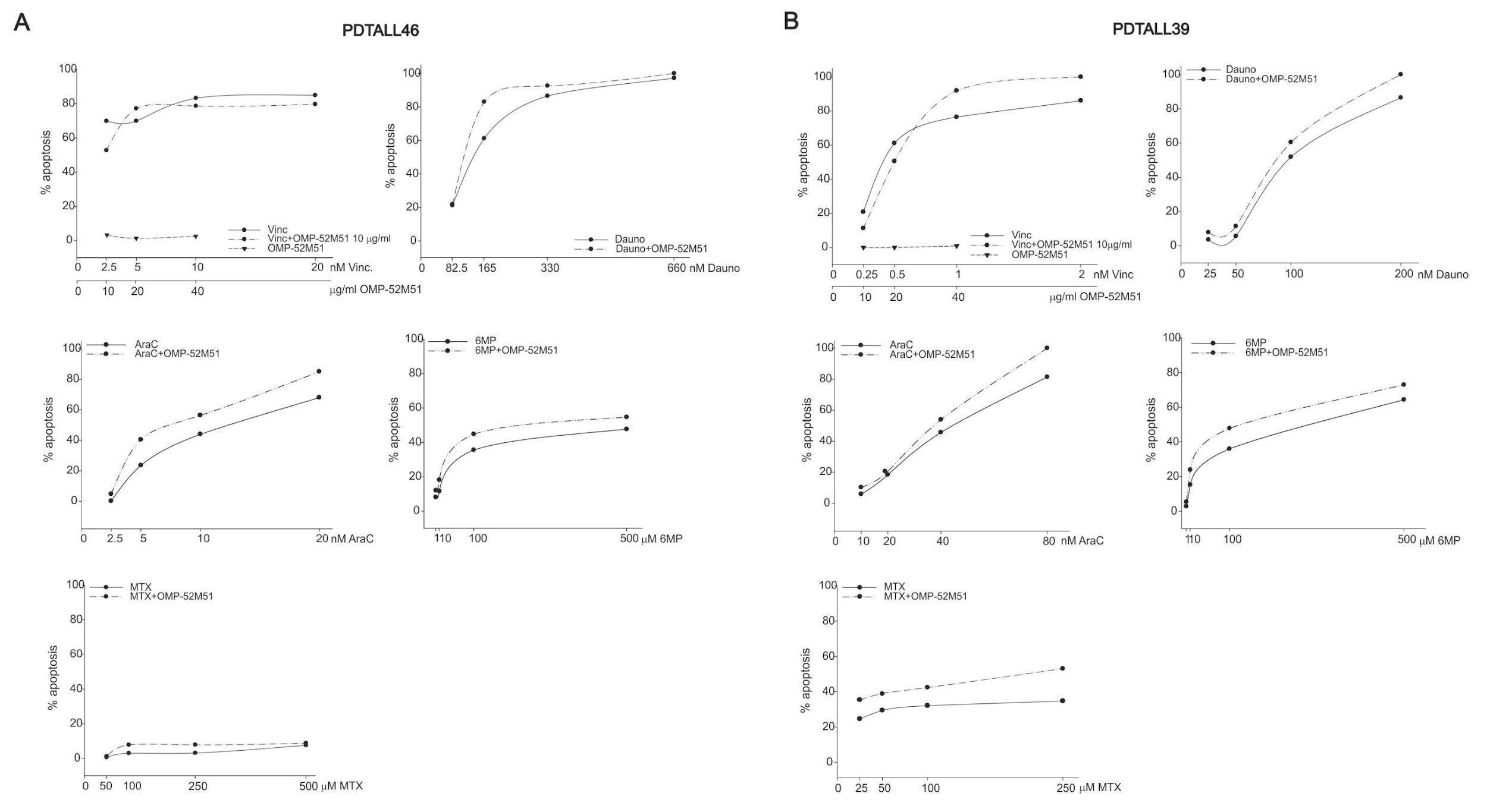


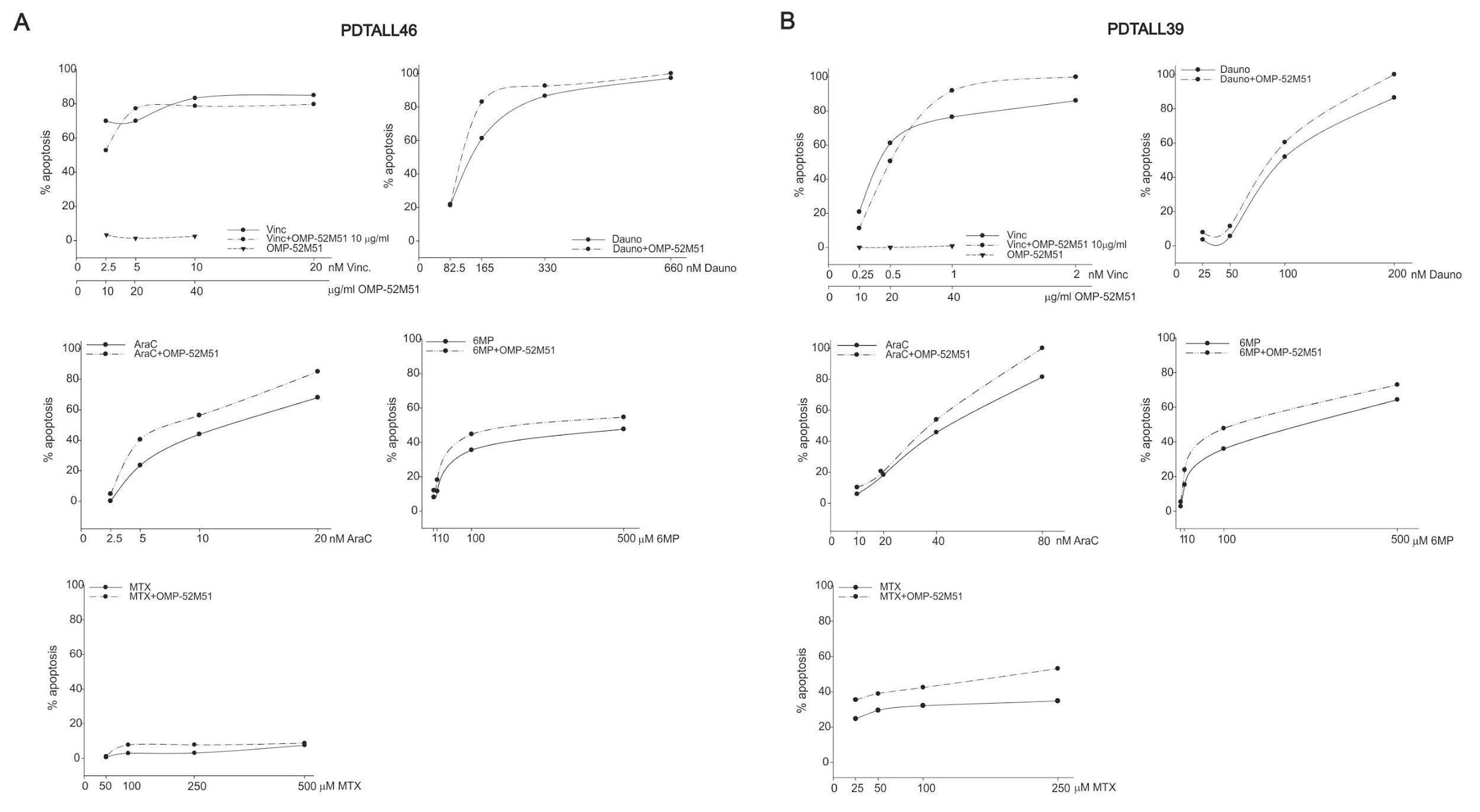


***In vitro* cell apoptosis determination in T-ALL PDXs cells treated with different drugs alone or in combination with OMP-52M51**

A-B. Dose–response curves of OMP-52M51 and its combination at constant concentration (10 μg/ml) with vincristine (Vinc), daunorubicin (Dauno), cytarabine (AraC), 6-mercaptopurine (6MP) and methotrexate (MTX) in PDTALL46 (A) and PDTALL39 (B) cells are shown. Cell apoptosis was determined by Caspase 3/7 assay after 24/72 h of drug exposure. One representative graph is shown of at least three independent experiments.

**Figure S5.**


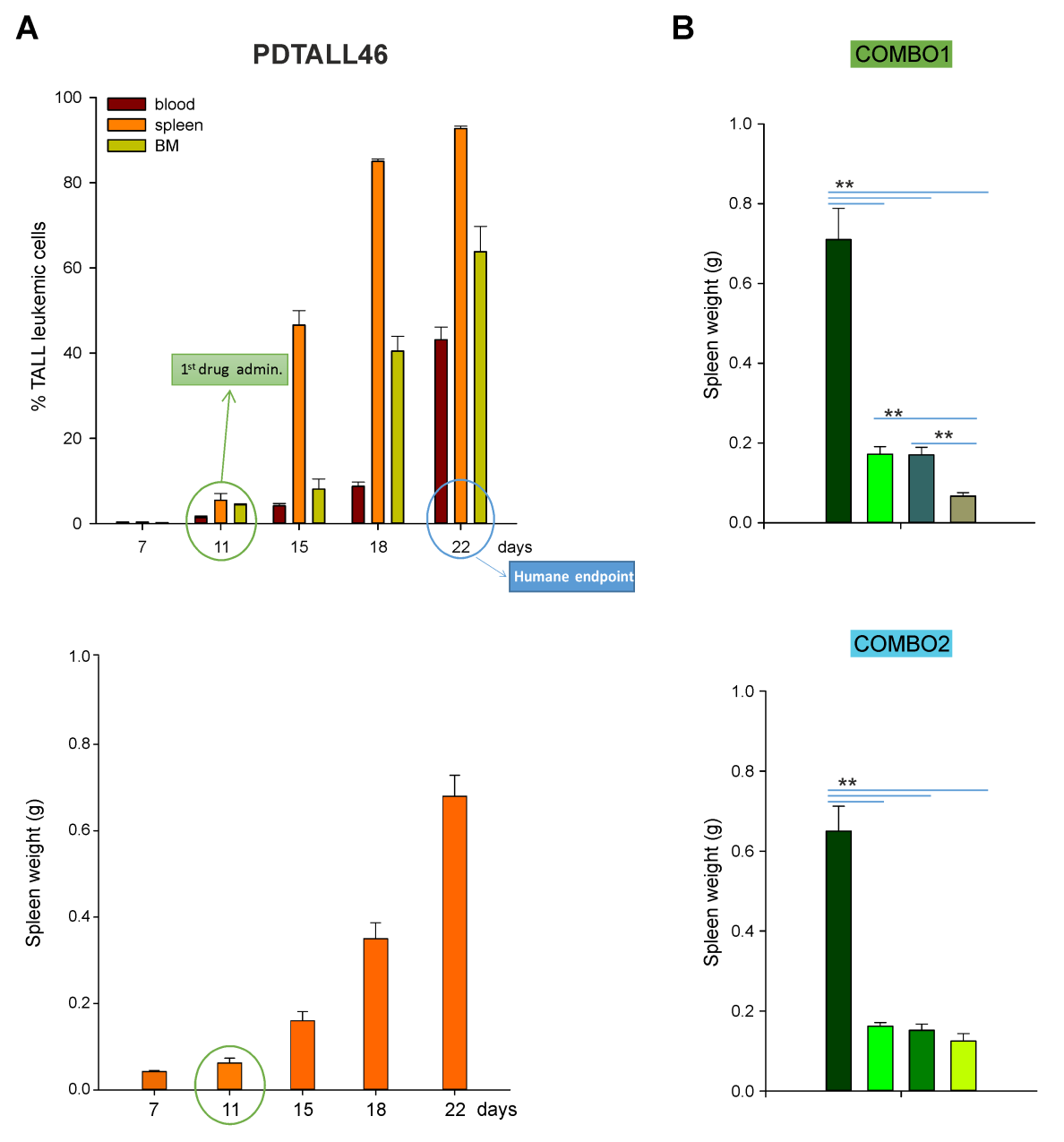


***In vivo* inhibitory effect of OMP-52M51 in combination with COMBO1 and COMBO2 in PDTALL46 model.**

A. Pilot experiment to evaluate the kinetics of leukemia engraftment of PDTALL46 cells in NSG mice. Top panel shows the percentage of human T-ALL cells in blood, spleen and BM of mice injected with leukemia cells at various time points and in the absence of any treatment. The circles indicate the time point selected for the first drug/antibody administration when leukemia is engrafted in blood, spleen end BM and the humane endpoint. Bottom panel shows the augment of spleen weight as a result of leukemia progression.

B. Therapeutic effects of COMBO1 (top panel) and COMBO2 (bottom panel) chemotherapy alone or in combination with OMP-52M51 of PDTALL outgrowth in NSG mice. Both panels indicate the weight of the spleens at sacrifice, which is used as a surrogate indicator of the leukemia burden.

Five to six mice per group were used. (*** P<0.01*).

**Figure S6.**

**
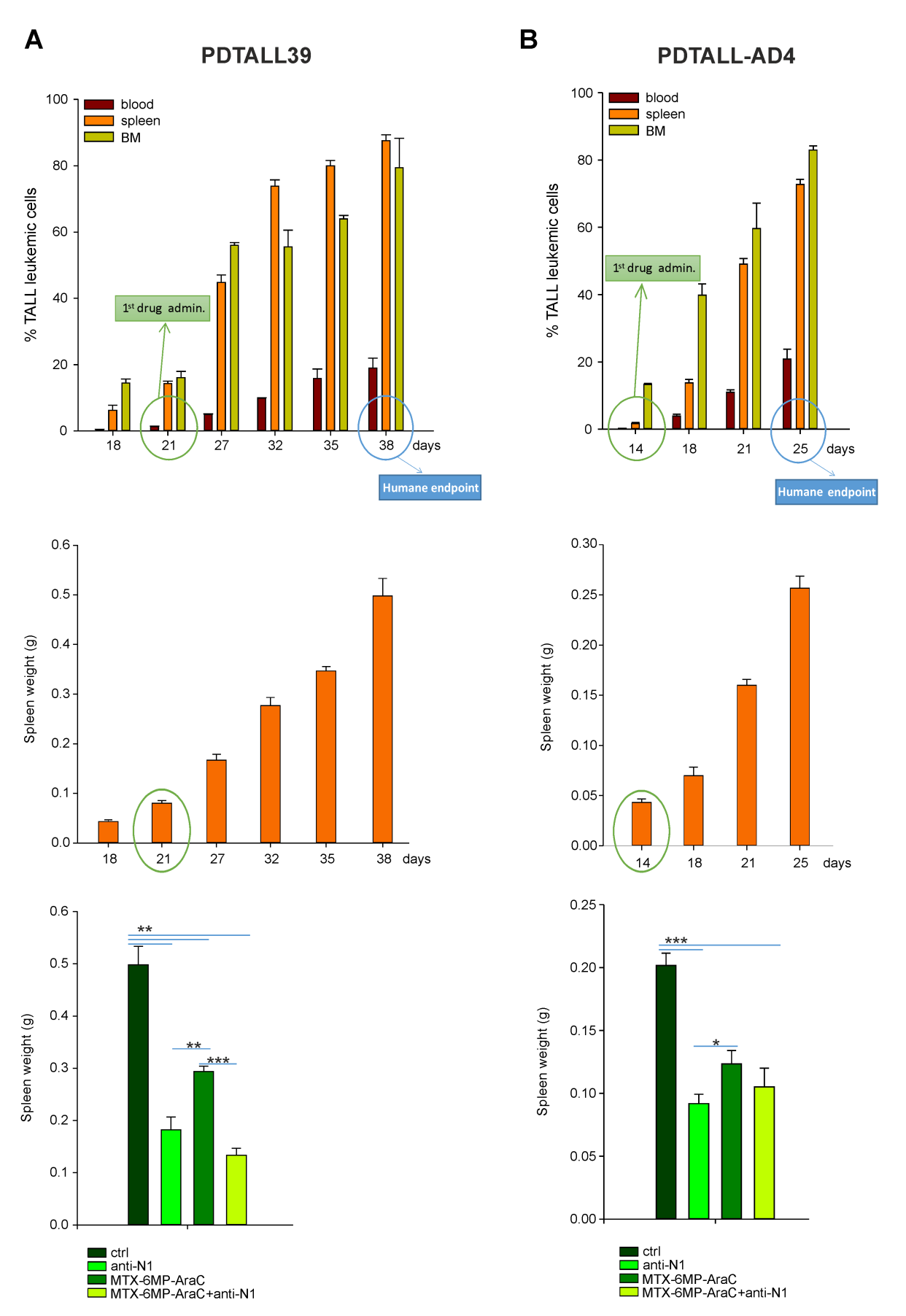
**

***In vivo* inhibitory effect of OMP-52M51 in combination with antimetabolite drugs (COMBO2) in PDTALL39 and PDTALL-AD4 models.**

A-B (top and middle panels). Pilot experiment to evaluate the kinetics of leukemia engraftment of PDTALL39 and PDTALL-AD4 cells in NSG mice. Top panels show the percentage of human T-ALL cells in blood, spleen and BM of mice injected with leukemia cells at various time points and in the absence of any treatment. The circles indicate the time point selected for the first drug/antibody administration when leukemia is engrafted in blood, spleen end BM and the humane endpoint. Middle panels show the augment of spleen weight as a result of leukemia progression.

A-B (bottom panels). Therapeutic effects of COMBO2 chemotherapy alone or in combination with OMP-52M51 of PDTALL39 and PDTALL-AD4 outgrowth in NSG mice. Bottom panel indicates the weight of the spleen at sacrifice, which is used as a surrogate indicator of the leukemia burden.

Five to six mice per group were used. (* *P<0.05; ** P<0.01;* **** P<0.001*).

**Figure S7.**

**
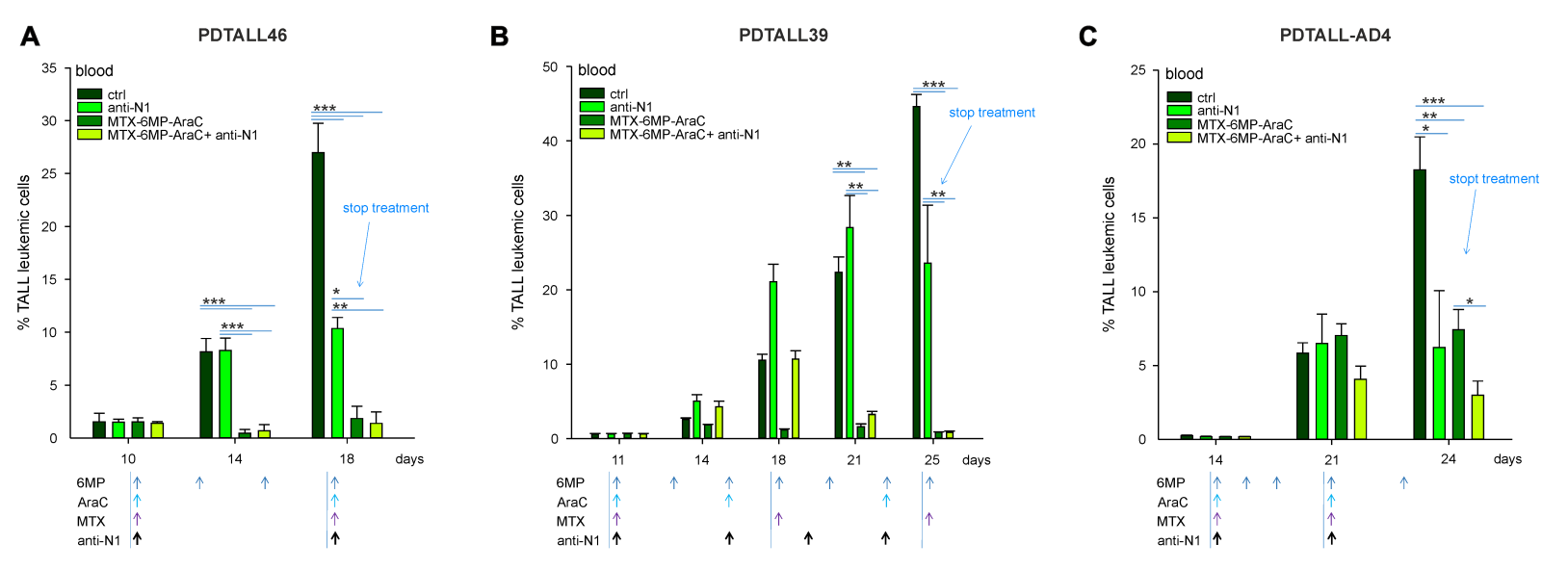
**

**Efficacy of Anti-NOTCH1 in combination with antimetabolite drugs in T-ALL PDXs models.** Effect of COMBO2 chemotherapy alone or in combination with OMP-52M51 in PDTALL46 (A), PDTALL39 (B) and PDTALL-AD4 (C) injected NSG mice. A-B-C, panels show the percentage of T-ALL cells in the blood of NSG mice at various time points till control group sacrifice, corresponding to treatment suspension. Four to seven mice per group were used (* *P<0.05; ** P<0.01;* **** P<0.001*).

**SUPPLEMENTARY REFERENCES**

1. Agnusdei V, Minuzzo S, Frasson C, et al. Therapeutic antibody targeting of Notch1 in T-acute lymphoblastic leukemia xenografts. *Leukemia*. 2014;28(2):278-88.
2. Lee EM, Yee D, Busfield SJ, et al. Efficacy of an Fc-modified anti-CD123 antibody (CSL362) combined with chemotherapy in xenograft models of acute myelogenous leukemia in immunodeficient mice. *Haematologica*. 2015;100:914-26.
3. Hartford C, Vasquez E, Schwab M, et al. Differential effects of targeted disruption of thiopurine methyltransferase on mercaptopurine and thioguanine pharmacodynamics. *Cancer Res.* 2007;67:4965-72.
4. Samuels AL, Beesley AH, Yadav BD, et al. A pre-clinical model of resistance to induction therapy in pediatric acute lymphoblastic leukemia. *Blood Cancer J.* 2014;4:e232.
5. Ramsey LB, Janke LJ, Edick MJ, e al. Host thiopurine methyltransferase status affects mercaptopurine antileukemic effectiveness in a murine model. *Pharmacogenet Genomics.* 2014;24:263-71.
6. Agnusdei V, Minuzzo S, Pinazza M, et al. Dissecting molecular mechanisms of resistance to NOTCH1-targeted therapy in T-cell acute lymphoblastic leukemia xenografts. *Haematologica*. 2020;105(5):1317-1328.
7. Bordin F, Piovan E, Masiero E, et al. *WT1* loss attenuates the TP53-induced DNA damage response in T-cell acute lymphoblastic leukemia. *Haematologica*. 2018;103(2):266-277.
8. Subramanian A, Tamayo P, Mootha VK, et al. Gene set enrichment analysis: a knowledge-based approach for interpreting genome-wide expression profiles. *Proc Natl Acad Sci U S A.* 2005;25;102(43):15545-50.
9. Mootha VK, Lindgren CM, Eriksson KF, et al. PGC-1alpha-responsive genes involved in oxidative phosphorylation are coordinately downregulated in human diabetes. *Nat Genet*. 2003;34(3):267-73.
